# Association of Lupus Anticoagulant with Brain Atrophy in Gulf War Illness (GWI)

**DOI:** 10.29245/2578-3009/2021/2.1215

**Published:** 2021-05-27

**Authors:** Lisa M. James, Peka Christova, Rachel A. Johnson, Brian E. Engdahl, Scott M. Lewis, Adam F. Carpenter, Apostolos P. Georgopoulos

**Affiliations:** 1 Brain Sciences Center, Department of Veterans Affairs Health Care System, Minneapolis, MN, 55417, USA; 2 Department of Neuroscience, University of Minnesota Medical School, Minneapolis, MN 55455, USA; 3 Department of Psychiatry, University of Minnesota Medical School, Minneapolis, MN 55455, USA; 4 Department of Psychology, University of Minnesota Medical School, Minneapolis, MN 55455, USA; 5 Department of Neurology, University of Minnesota Medical School, Minneapolis, MN 55455, USA

**Keywords:** Lupus anticoagulant, Brain volume, Gulf War Illness, Antiphospholipid antibodies, Autoimmunity

## Abstract

Separate lines of research have documented brain atrophy and evidence of autoimmune mechanisms in Gulf War Illness (GWI), including the presence of lupus anticoagulant (LAC), in veterans with GWI. Here we evaluated the possible association of LAC and brain volume in veterans with GWI. The presence of LAC was determined using Silica Clotting Time and dilute Russell’s Viper Venom Time assays. MRI data was acquired using a Philips 3T MR scanner from which total gray matter, total cortical gray matter, total subcortical gray matter, and total cerebral white matter were derived. The results demonstrated a statistically significant reduction of brain volume in all regions tested in GWI veterans with positive LAC, as compared to those without LAC. These findings add to the literature implicating autoimmune mechanisms in GWI and point to the presence of prothrombotic antiphospholipid antibodies as contributing to brain atrophy in GWI.

## Introduction

One-third of U.S. Veterans of the 1990–91 Persian Gulf War are affected by Gulf War Illness (GWI), a condition characterized by chronic and often disabling symptoms involving multiple organ systems including, prominently, the brain^1,2^. Numerous brain abnormalities have been associated with GWI^3–9^. For instance, several recent studies have documented brain atrophy in veterans with GWI^7–11^. In addition, brain functional anomalies in veterans with GWI have been shown to be indistinguishable from those of known autoimmune disorders^6^, supporting theories that GWI may, in part, be related to autoimmunity^12–14^. Providing further support for autoimmune processes in GWI, we recently reported lupus anticoagulant (LAC) positivity in one-quarter of veterans with GWI, a rate that was comparable to that found in veterans diagnosed with well-established autoimmune disorders^15^. LAC, an indicator of autoimmunity, is an autoantibody against phospholipids or phospholipid-binding proteins that results in formation of blood clots in vivo. LAC has been associated with ischemic brain changes including brain atrophy in systemic lupus erythematosus (SLE)^16^ and neuropsychiatric SLE^17^. Here we evaluated the association of LAC and brain volume in veterans with GWI.

## Methods

Sixty-eight veterans (64 men) who met criteria for GWI and no diagnosed autoimmune conditions participated in this study as paid volunteers (see ref^8^ for GWI diagnostic criteria). This sample consisting of veterans studied using magnetic resonance imaging (MRI) is a subset of a larger one of 84 veterans studied for Lupus Anticoagulant (LAC) positivity^15^. The Minneapolis VA Health Care System (VAHCS) Pathology and Laboratory Medicine Service evaluated blood samples for markers related to inflammation and autoimmunity including C-reactive protein (CRP), C3, C4, platelet count, and LAC. LAC was assessed using a standard Lupus Inhibitor Panel (VA Document ID: HEM02–023, issue date: 11/4/2019) consisting of Silica Clotting Time (SCT) and dilute Russell’s Viper Venom time (dRVVT), with both screen and confirm tests being run simultaneously for both tests. Cutoff values were 1.19 for SCT C test ratio and 1.15 for dRVVT C test ratio; the Lupus Inhibitor Panel outcome was Negative if both values were below cutoffs and Positive if either value was above the cutoff. MRI data was acquired using a Philips 3T MR scanner (as detailed in ref^8^) from which we derived the volumes of the following regions using Freesurfer: total gray matter, total cortical gray matter, total subcortical gray matter, and total cerebral white matter. The study was approved by the University of Minnesota and Minneapolis VA Health Care System Institutional Review Boards. Standard statistical methods including descriptive statistics (mean, SEM, etc.) and analysis of covariance (ANCOVA) were used to analyze the data in IBM-SPSS (version 26). Separate ANCOVAs were performed for each brain measure.

## Results

**Figure 1.**
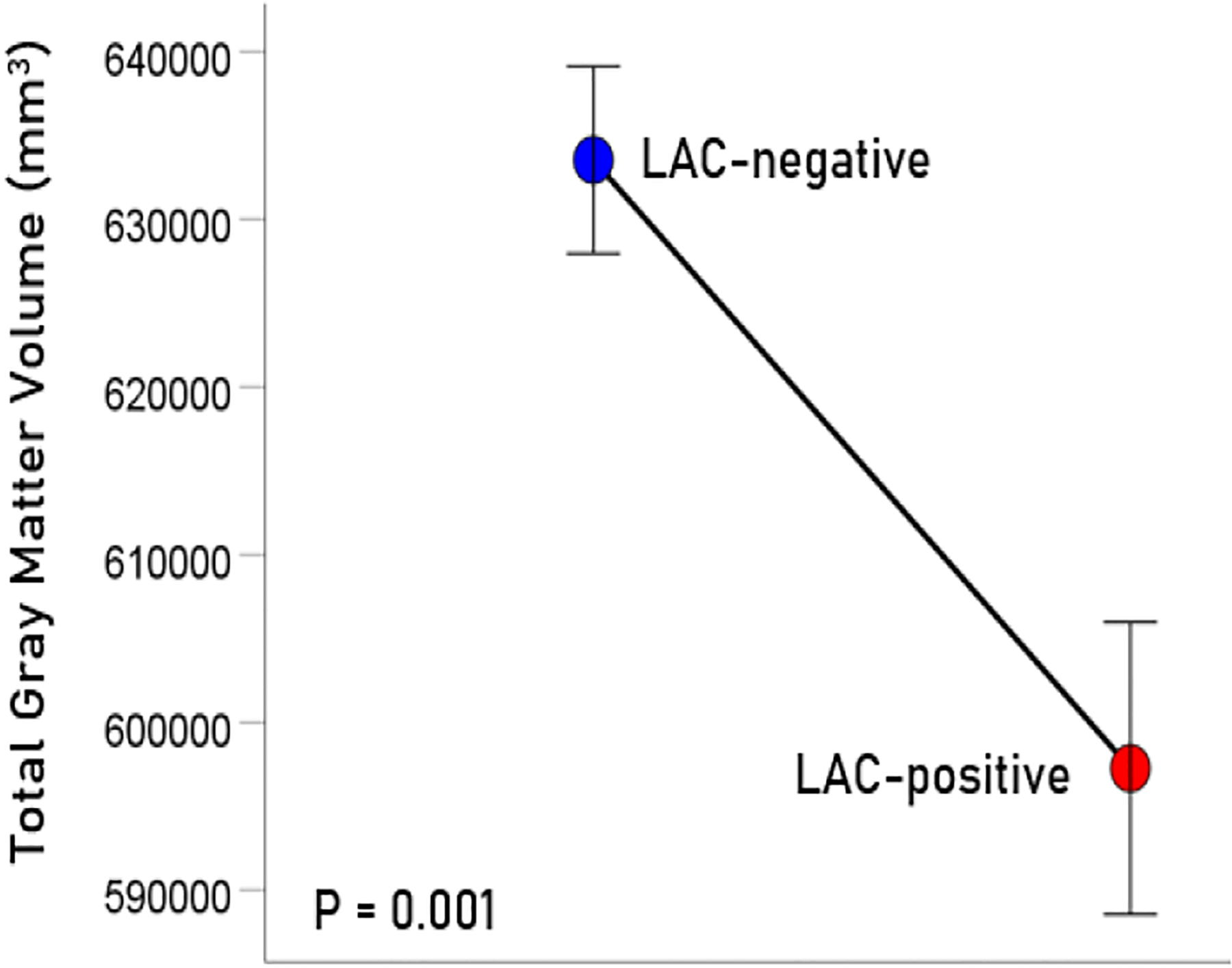
Mean (± SEM) of total gray matter volume for the LAC-negative and LAC-positive groups. Means are adjusted for total intracranial volume, age and gender (ANCOVA).

**Figure 2.**
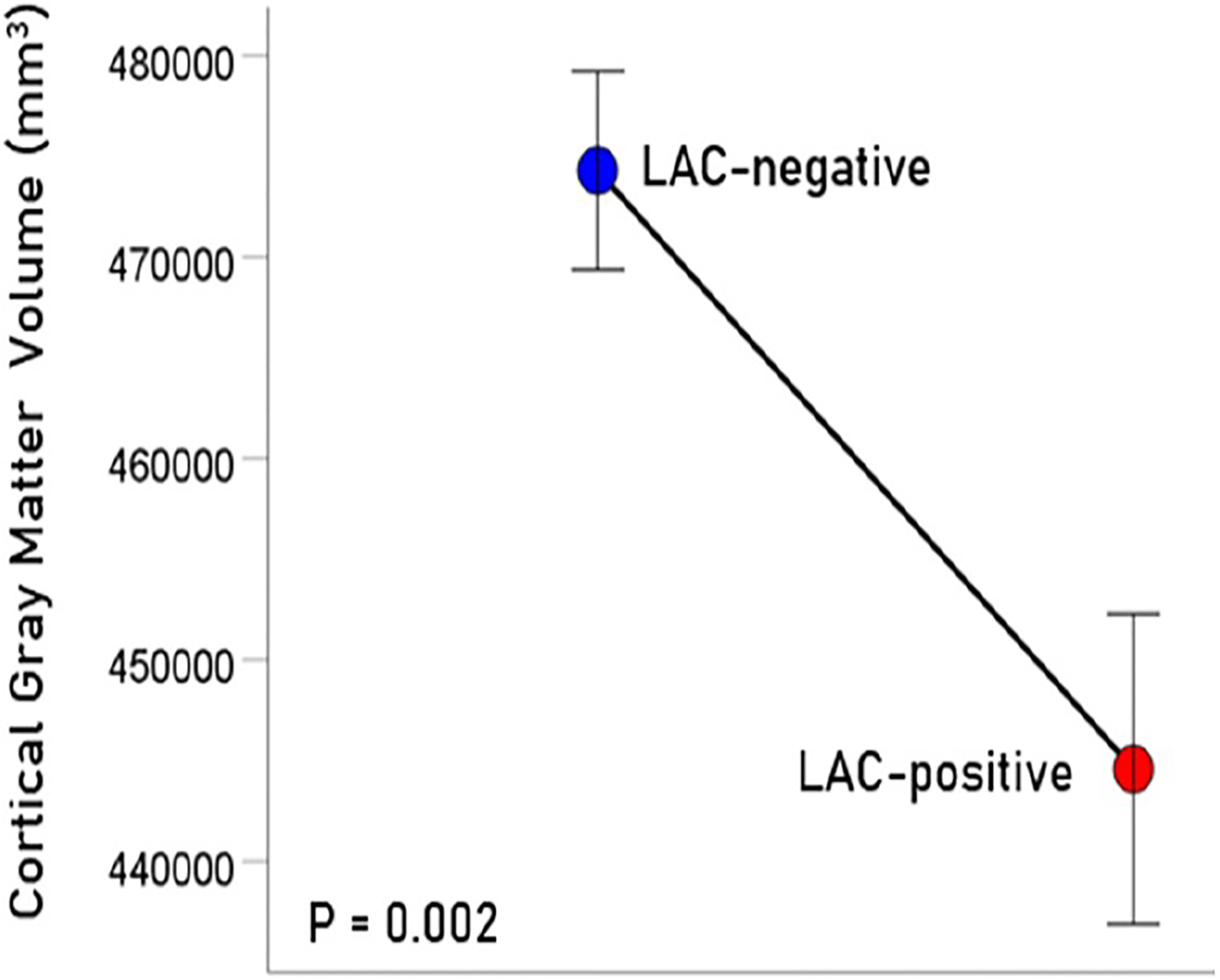
Mean (± SEM) of cortical gray matter volume for the LAC-negative and LAC-positive groups. Means are adjusted for total intracranial volume, age and gender (ANCOVA).

**Figure 3.**
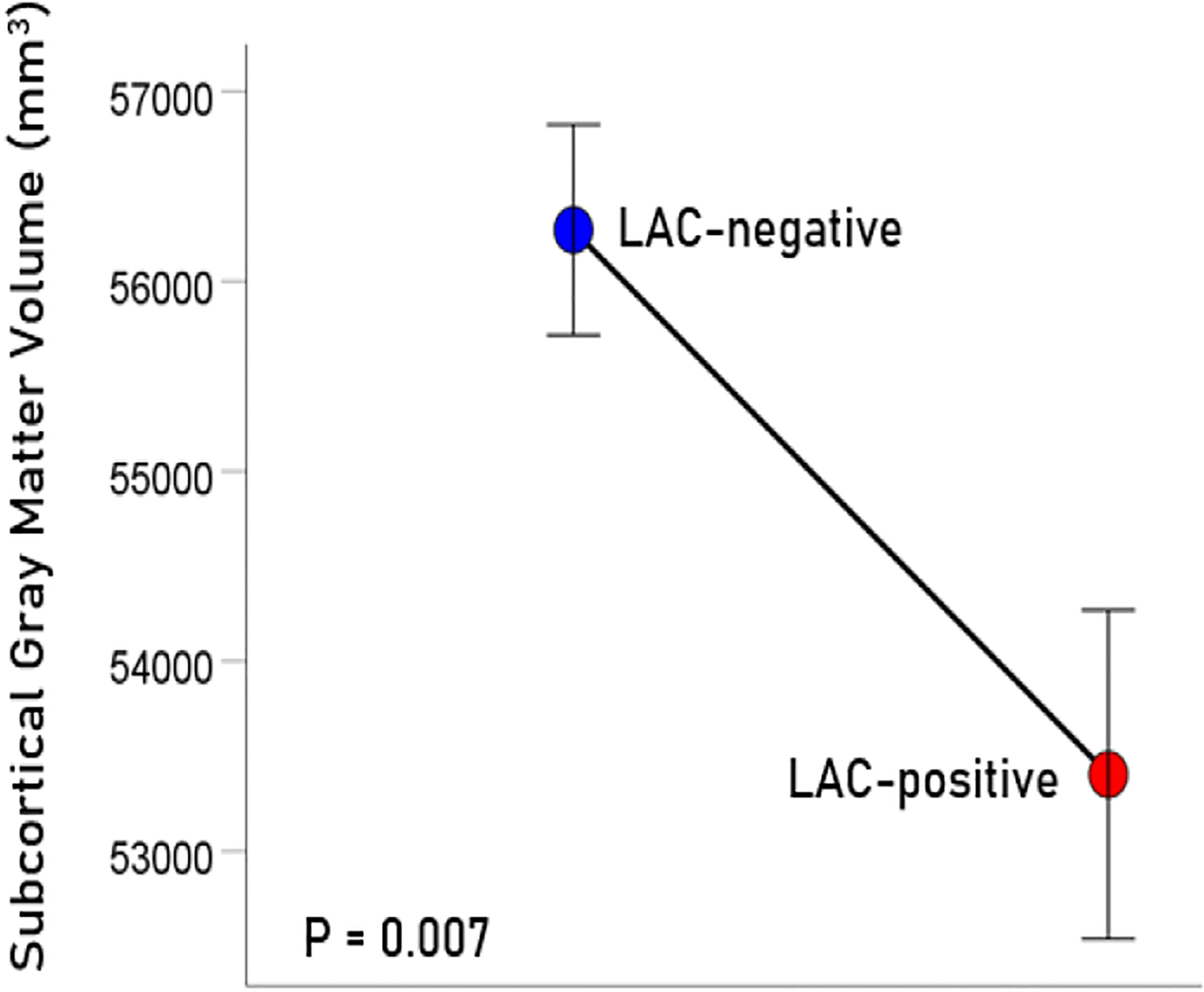
Mean (± SEM) of subcortical gray matter volume for the LAC-negative and LAC-positive groups. Means are adjusted for total intracranial volume, age and gender (ANCOVA).

**Figure 4.**
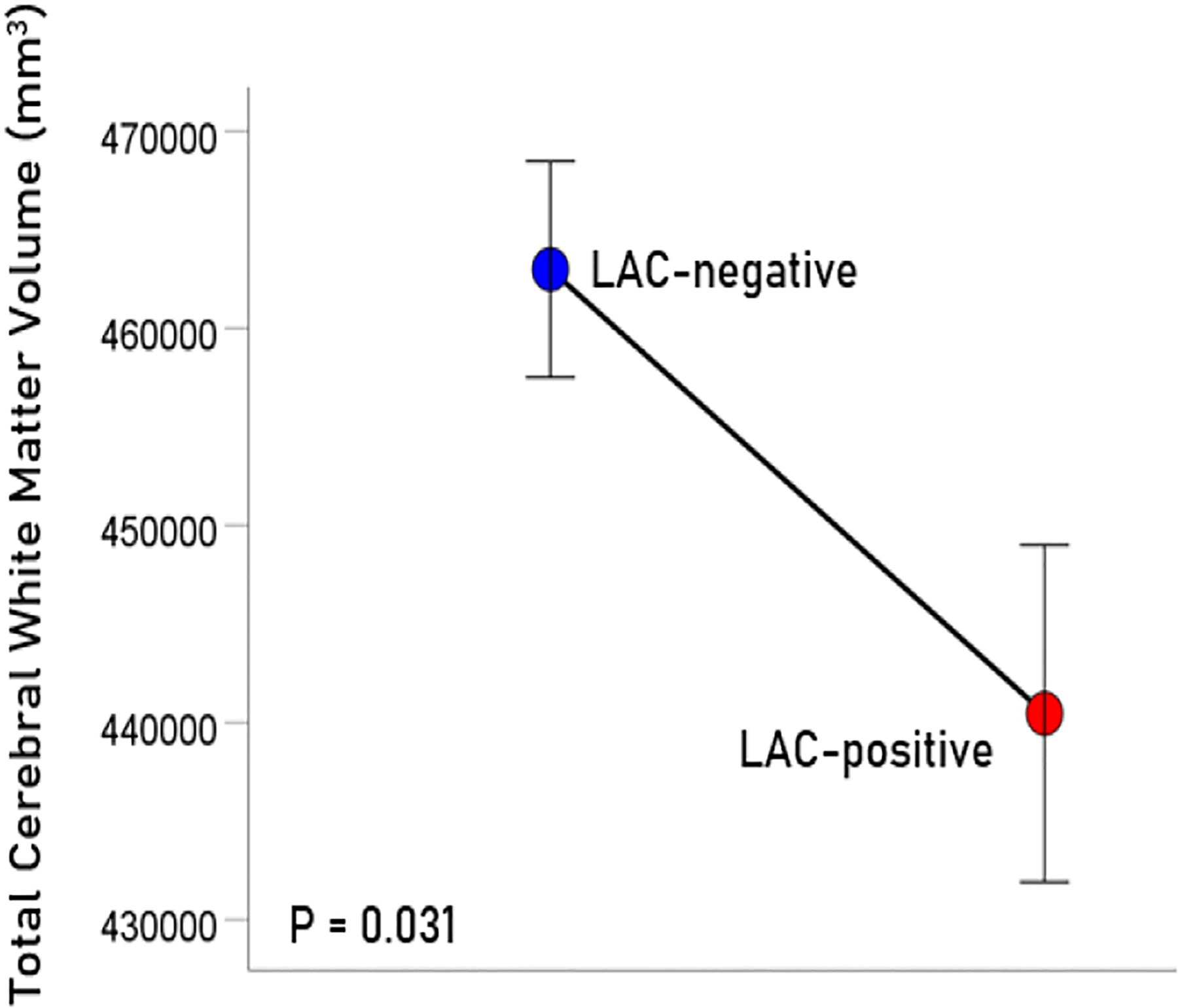
Mean (± SEM) of total cerebral white matter volume for the LAC-negative and LAC-positive groups. Means are adjusted for total intracranial volume, age and gender (ANCOVA).

The age (mean ± SEM) was 56.39 ± 1.15 for men (N = 64) and 48.87 ± 2.5 for women (N = 4); these means did not differ significantly (t_[66]_ = 1.61, P = 0.11). LAC was present in 20/68 (29.4%; all men) participants in this sample. This percentage is similar to those found in veterans with relapsing remitting multiple sclerosis (33.3%), rheumatoid arthritis (33.3%), Sjögren’s syndrome (12.5%) or lupus (28.6%)^15^. Age did not differ significantly between the LAC− and LAC+ groups (mean ± SEM: LAC-absent, 56.41 ± 1.39, N = 40; LA-present, 54.82 ± 1.76, N = 28; t_[66]_ = 0.652, P =0.517). A multivariate ANCOVA was used to evaluate the association of LAC and each brain volume measure, with total intracranial volume, age, and gender as covariates. (An initial analysis showed that CRP, C3, C4, and platelet count did not have a significant effect on any of the measures of brain volume and were not used in subsequent tests.) There was a statistically significant reduction of brain volume in the LAC+ group in all regions tested (Figures 1–4), by an average of 5.7% (Table 1).

## Discussion

Here we evaluated the association of lupus anticoagulant and brain volume in veterans with GWI and found that brain volume was significantly reduced in GWI veterans with positive lupus anticoagulant compared to those without lupus anticoagulant. These findings add to the literature implicating autoimmune mechanisms in GWI and suggest a potential role for prothrombotic antiphospholipid autoantibodies in GWI-related brain atrophy.

To our knowledge, this is the first study to link brain atrophy in GWI to the presence of antiphospholipid antibodies; however, antiphospholipid antibodies have been associated with several brain abnormalities in other conditions. LAC, in particular, has been associated with evidence of small vessel disease including white matter hyperintensities, microhemorrhage, and cortical atrophy in patients with neuropsychiatric SLE^17^. Similarly, LAC, but not other antiphospholipid antibodies, has been found to be associated with white matter lacunar infarcts and atrophy in patients with SLE^16^. In addition, LAC has been associated with cognitive dysfunction^18^ in patients with SLE and with neuropsychiatric manifestations^19^ of SLE. Likewise, antiphospholipid syndrome, which is defined in part by the presence of antiphospholipid antibodies including LAC, has also been widely associated with similar neuroimaging abnormalities including infarcts, white matter hyperintensities, atrophy, and microstructural white matter abnormalities^20^ as well as cognitive dysfunction^21^. Although the mechanisms of brain involvement in antiphsopholipid syndrome and SLE are not well understood, evidence supports a role of vasculopathy^22–24^, thrombosis^25–26^, and/or direct binding of antiphospholipid antibodies to neurons and glia^20^. The mechanisms underlying the association of LAC and brain atrophy in veterans with GWI remain to be elucidated. Furthermore, the cause of LAC positivity in GWI veterans is unclear although antiphospholipid antibodies typically occur as a result of molecular mimicry between phospholipid proteins and those of foreign antigens (e.g., infections, vaccines)^27–29^.

The present findings shed new light on brain abnormalities in GWI and support a role of autoimmune mechanisms in GWI-related atrophy. The findings, however, must be considered within the context of study limitations including a relatively small sample of predominantly male GWI veterans, absence of a control group, a cross-sectional study design, and evaluation of antiphospholipid antibody concentrations at a single time point. Longitudinal studies in larger samples of Gulf War veterans will be beneficial for evaluating the long-term impact of LAC-positivity on brain structure and function in veterans with and without GWI.

## Conclusion

This study documents autoimmune-associated brain atrophy in veterans with GWI, providing further evidence of GWI as a neuroimmune condition^6^. In addition to the brain effects observed here and elsewhere^16–21^, LAC is a risk factor for prothrombotic events^30^ and myocardial infarction^31^, both of which occur at significantly higher rates in male Gulf War veterans compared to the general population^32^. Thus, it may be prudent to monitor Gulf War veterans for evidence of hypercoagulation to reduce further deleterious health effects associated with LAC-positivity.

## Figures and Tables

**Table 1:** Statistics for measures and comparisons between LAC groups. Means are adjusted for age, gender and total intracranial volume.

Region	LAC negative (N = 40)		LAC positive (N = 28)		Group comparisons (LAC positive) – LAC negative)	
	Mean (mm^3^)	SEM	Mean (mm^3^)	SEM	% change	P-value (F-test, ANCOVA)
Total gray matter	633541	5574	597276	8708	−5.72%	0.001
Cortical gray matter	474296	4929	444581	7700	−6.26%	0.002
Subcortical gray matter	56271	554	53404	866	−5.10%	0.007
Cerebral white matter	462993	5474	440461	8552	−4.87%	0.031
